# Inhibitors of Sodium‐Dependent Serotonin Transporter Protein (SERT) as Potential New Nematicides

**DOI:** 10.1002/cbdv.202503443

**Published:** 2026-03-26

**Authors:** Geraldo Jamisse Hodela, Vitor Pereira de Sousa, Mariana Castro de Melo, Rodrigo Martins Fráguas, Willian César Terra, Denilson Ferreira de Oliveira

**Affiliations:** ^1^ Department of Chemistry Federal University of Lavras Lavras MG Brazil; ^2^ Department of Phytopathology Federal University of Lavras Lavras MG Brazil

**Keywords:** *Meloidogyne*, molecular docking, nematicide, paroxetine, sodium‐dependent serotonin transporter

## Abstract

Nematodes of the genus *Meloidogyne* cause major losses in agricultural production worldwide. To identify potentially useful chemical structures for developing new nematicides, this study initially aimed to determine in silico  – using computationally efficient techniques – the protein target of chaetoglobosins A and B in these nematodes. This process led to the selection of the sodium‐dependent serotonin transporter protein (SERT). The activities of SERT inhibitors were subsequently evaluated in vitro. The best result was obtained with paroxetine, which caused 50% mortality (LC_50_) in second‐stage juveniles (J2) of *Meloidogyne incognita* at a concentration of 351.3 *µ*g/mL. Under the same conditions, the commercial nematicide fluensulfone showed an LC_50_ of 39.3 *µ*g/mL. In plant trials, paroxetine reduced the pathogenicity of *M. incognita* J2. Therefore, further investigation of SERT inhibitors holds promise for the development of new nematicides.

## Graphical abstract

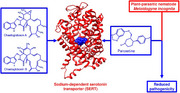

## 1 Introduction

**FIGURE 1:**
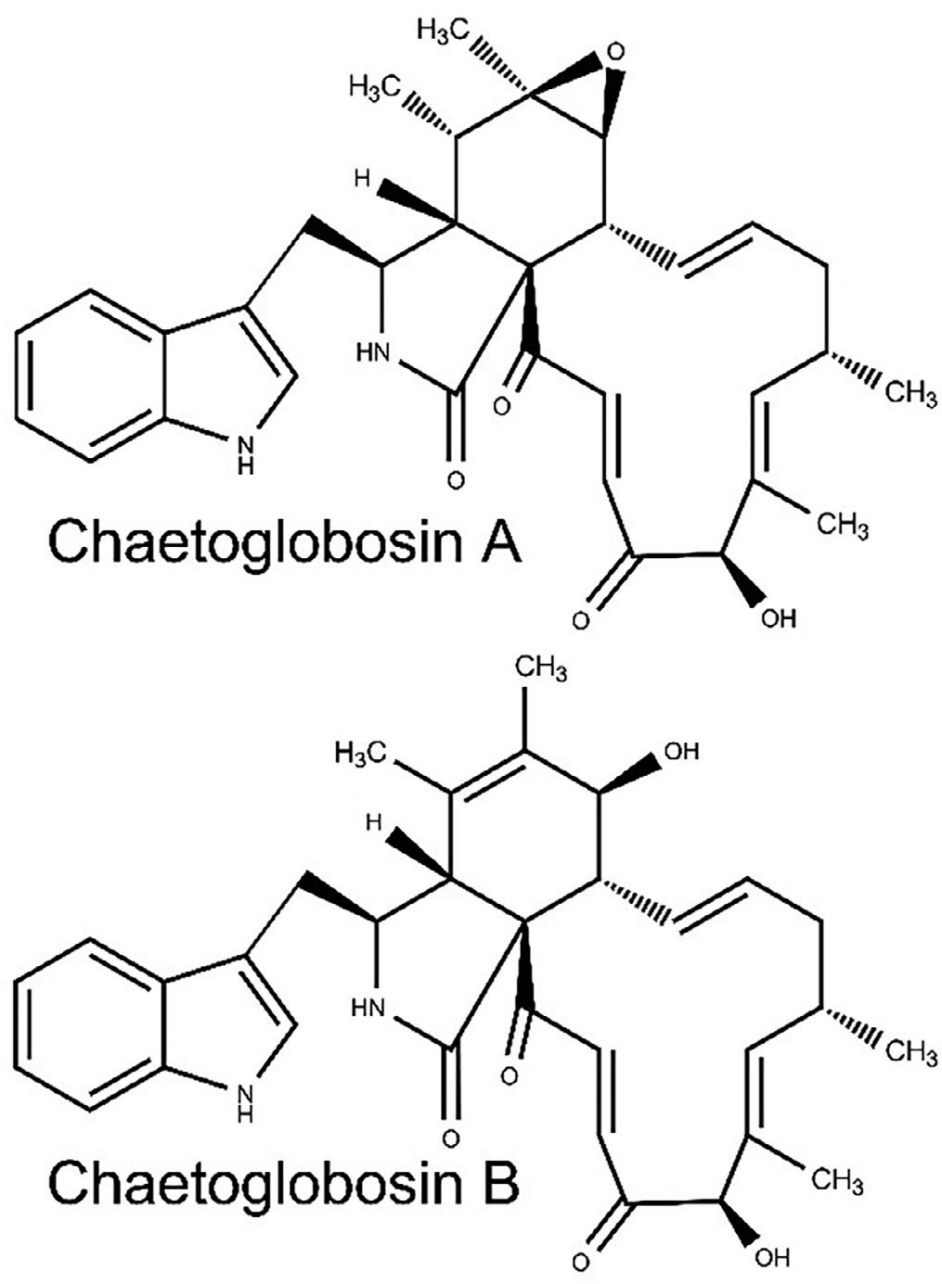
Chemical structures of chaetoglobosins.

Plant‐parasitic nematodes (PPNs) significantly reduce agricultural production worldwide [1]. Among these soil‐borne pathogens are the root‐knot nematodes (RKNs, *Meloidogyne* spp.), which cause damage across various regions of the world [2]. Currently, chemical nematicides remain one of the primary methods for controlling RKNs; however, they pose risks to both the environment and human health [3]. As a result, many chemical nematicides have been gradually withdrawn from the market [2, 4], increasing the demand for new products for RKNs control. Consequently, new compounds [5] – particularly those of natural origin – have been tested against RKNs [6, 7, 8]. One such example is chaetoglobosins A and B (Figure 1). According to the literature [9], the lethal concentration required to affect 50% (LC_50_) of second‐stage juveniles (J2) of *Meloidogyne javanica* (Treub) Chitwood was 88.40 *µ*g/mL for chaetoglobosin A and 107.07 *µ*g/mL for chaetoglobosin B. In a plant inoculation experiment using the same nematode species, researchers found that chaetoglobosins A and B reduced the number of galls (nematode pathogenicity) by 59%–61.5%, the number of egg masses by 71.1%–72.4%, and the number of eggs by 30.1%–35.4%.

Although chaetoglobosins have shown promising results, their structural complexity makes large‐scale production for commercial RKNs control economically unfeasible at present. However, there is potential for the use of chaetoglobosins as a starting point for the development and/or discovery of organic compounds with simpler chemical structures, but still with nematicidal activity. To address this challenge, understanding how chaetoglobosins interact with RKNs could facilitate the development of simpler, effective compounds. With this goal in mind, the primary objective of this study was to identify in silico the protein target of chaetoglobosins in *Meloidogyne* spp. using computationally efficient techniques, and evaluate the nematicidal activities of inhibitors of the identified enzyme. The specific objectives were as follows: (i) select protein ligands structurally similar to chaetoglobosins A and B; (ii) identify, among the proteins whose ligands were selected, those with the greatest similarity in amino acid sequences to those of *Meloidogyne* spp.; (iii) conduct molecular docking of the chaetoglobosins and inhibitors of the selected proteins to determine which protein sites could be inhibited by chaetoglobosins; and (iv) perform biological tests with *M. incognita* and inhibitors of the selected proteins to validate the results obtained in silico.

## 2 Results and Discussion

### 2.1 Preparation of Three‐Dimensional Structures of Chaetoglobosins

Two conformations were observed to be the most stable for each of the two substances (Figure S1). For chaetoglobosin A, an RMSD of 2.45 Å was observed between conformations 2 and 42, while chaetoglobosin B exhibited an RMSD of 1.96 Å between conformations 38 and 40. Therefore, both conformations for each chaetoglobosin were considered in subsequent steps.

### 2.2 Protein Ligands Three‐Dimensionally Similar to Chaetoglobosins

The search for three‐dimensional structures similar to chaetoglobosins identified ligands for the following proteins: sodium‐dependent serotonin transporter (SERT) [10], glutaminase [11], and AmpC*β*‐lactamase [12] (Table S1). These findings suggest that chaetoglobosins A and B may also inhibit these proteins.

### 2.3 Amino Acid Sequence Analysis

In the search for amino acid sequences similar to the three selected proteins contained within the genomes of *Meloidogyne* spp. (taxid:18290), the only protein with a score above 200 was the SERT [10]. Its score, identity, and coverage were 610%, 53.28%, and 81%, respectively (Table S2), suggesting that SERT may be a target of chaetoglobosins in *Meloidogyne* spp.

SERT is a protein encoded by the *SLC6* gene and functions as a neurotransmitter‐related transporter, facilitating the movement of serotonin, sodium, and chloride from the synaptic spaces to the presynaptic neurons. In this process, SERT also transports potassium ions out of the cells. Located in cell membranes, it consists of approximately 630 amino acid residues [13, 14]. In nematodes, serotonin plays an important role in host‐plant invasion by inducing rhythmic stylet movement [15] and influencing egg‐laying behavior [16]. One example is research involving reserpine, an alkaloid isolated from *Rauwolfia serpentina* L. [15], which inhibited serotonergic signaling, negatively affecting the nematode's ability to invade the host plant. Similarly, a study on the nematicidal activity of bilobalide against *Caenorhabditis elegans* Maupas demonstrated its inhibitory effect on nematode egg‐laying [16]. These findings suggest that substances inhibiting SERT may serve as promising candidates for the development of new nematicides to control PPNs.

### 2.4 Molecular Docking

**FIGURE 2:**
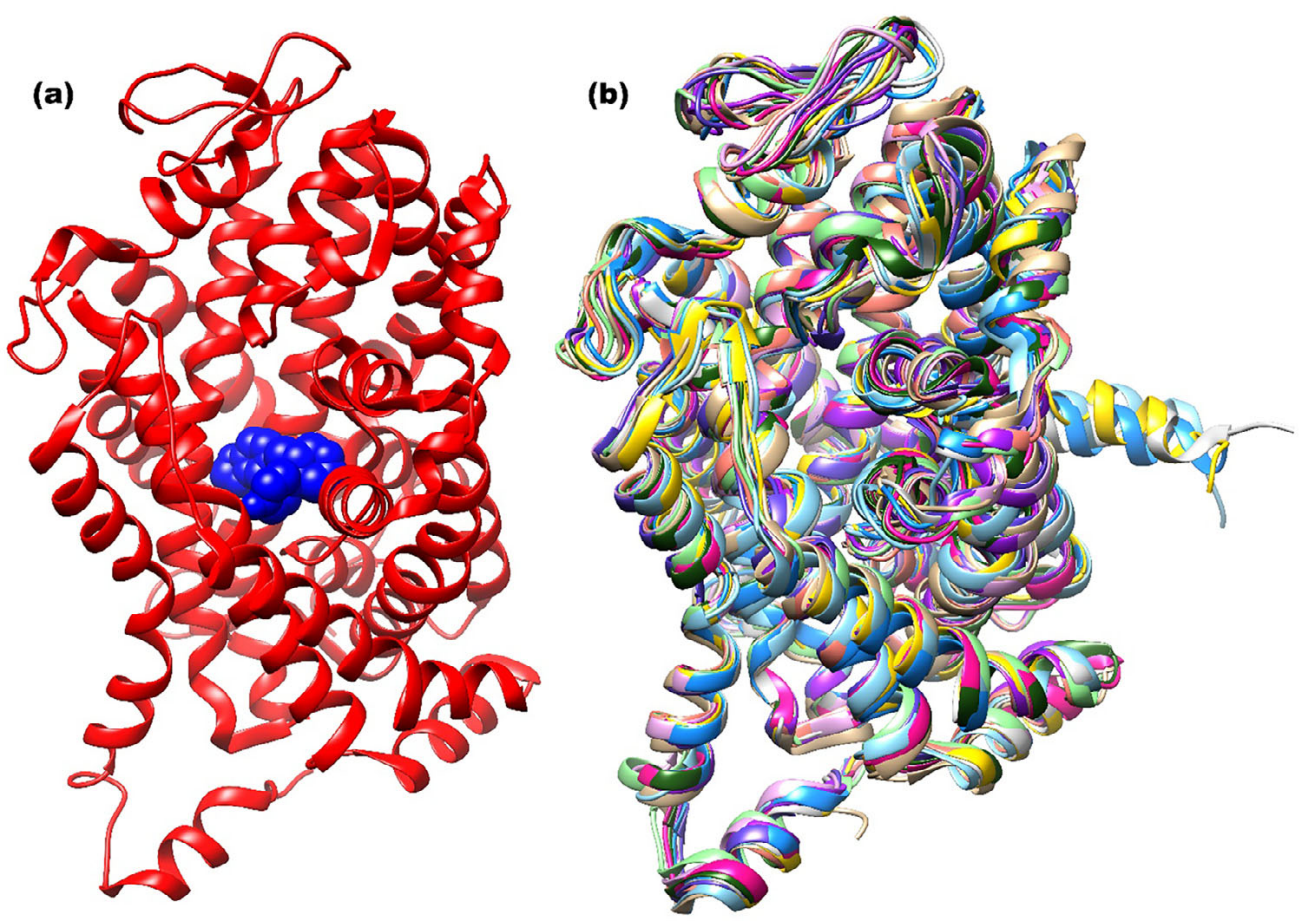
(a) Three‐dimensional structure of the sodium‐dependent serotonin transporter (SERT) 6VRH [17], with the ligands experimentally complexed to various SERTs in blue; (b) Three‐dimensional structures of sodium‐dependent serotonin transporters 7MGW [18], 7LI7 [18], 7LWD [19], 7LIA [18], 7LI9 [18], 7LI8 [18], 7LI6 [18], 6DZZ [20], 6VRH [17], 6VRL [17], 6VRK [17], and 6DZV [17] aligned by the Lovoalign 18.320 program [21]. Image generated by UCSF Chimera 1.13.1 [22].

**FIGURE 3:**
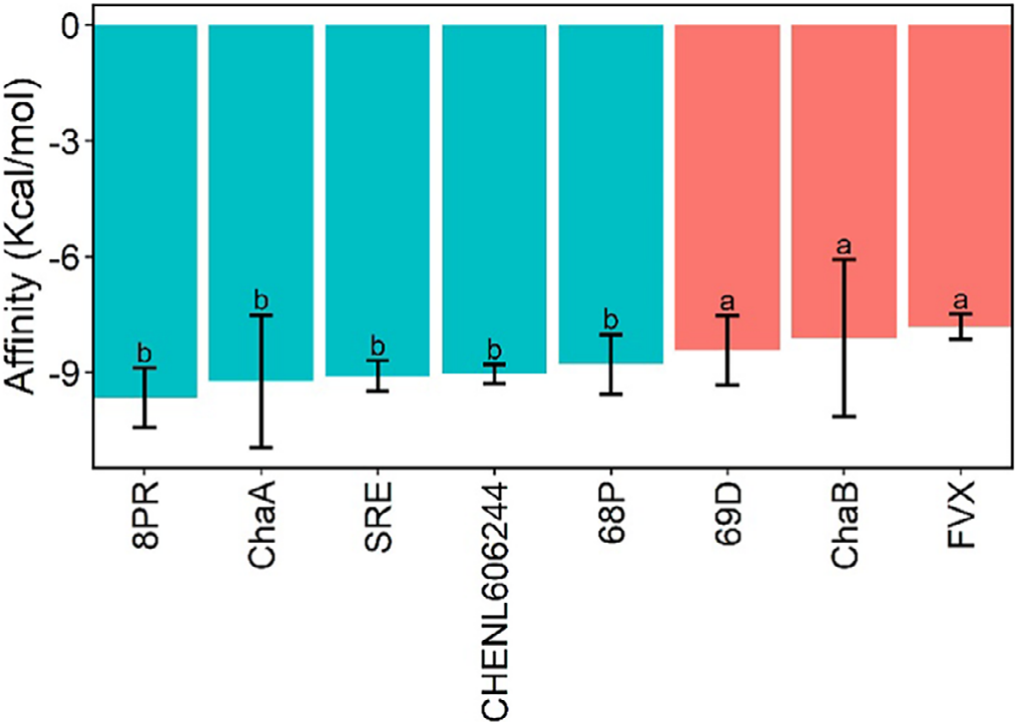
Affinities of chaetoglobosins A (ChaA) and B (ChaB), and of the following inhibitors of sodium‐dependent serotonin transporters (SERTs): 68P [23], 69D [23], 8PR (Paroxetine) [17], CHEMBL606244 [10], FVX [24], and SRE [24]. The affinities for the SERTs active site were calculated using the QuickVina 2 [25] computer program. Columns with the same letter are statistically equal to each other according to the Scott‐Knott test [26] (*p* < 0.05; n = 56). Error bars correspond to the standard deviation.

Both the active sites of the SERTs used (Figure 2a) and their overall 3D structures (Figure 2b) showed strong overlap, indicating favorable conditions for obtaining data with normality and homoscedasticity during the molecular docking stage. These properties were confirmed by statistical calculations, which also demonstrated that the affinities of the chaetoglobosins for SERTs were statistically equivalent to the affinities calculated for SERT inhibitors (Figure 3). When the 3D structure of the ligand 8PR (paroxetine), obtained by docking to the SERT 6VRH [17] was compared to the 3D structure of the same ligand experimentally complexed to the same SERT, the RMSD value was observed to be 0.3 Å. This suggests that the docking process adequately reproduces the experimental data. As for the ligands SRE, 68P, 69D, and FVX (Figure 3), which were experimentally complexed to the SERTs 6AWQ, 5I73, 5I74, and 6AWP, respectively, the same comparison was not possible, since these SERTs were mutants and/or had missing amino acid residues in their binding sites, which made their use in the molecular docking process impossible. These findings support the hypothesis that SERT is the target of the chaetoglobosins in *Meloidogyne* spp.

**FIGURE 4:**
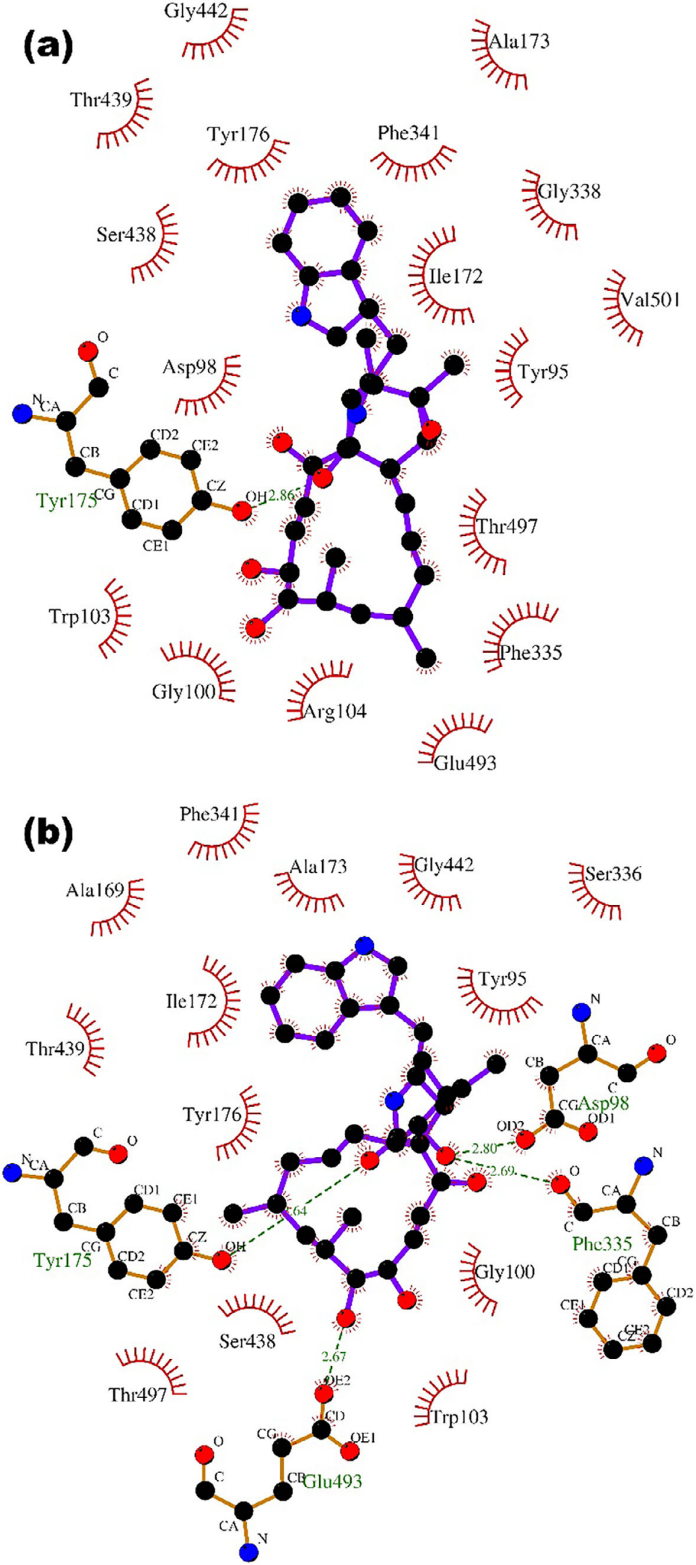
(a) Two‐dimensional representation of the interactions of chaetoglobosin A with the active site of the sodium‐dependent serotonin transporter 6VRL [17], to which the compound was docked with the computer program QuickVina 2 [25]. (b) The same representation for chaetoglobosin B. This figure was generated with the program LigPlot 2.2.8 [27].

When analyzing the interactions of chaetoglobosins with the active site of SERTs, hydrogen bonds were formed from the oxygen atoms from different organic functions (Figure 4). The amino acid residues Tyr (175), Glu (493), Asp (98), and Phe (335) were responsible for the interaction with chaetoglobosin B (Figure 4b), while chaetoglobosin A formed a hydrogen bond with the Tyr (175) residue (Figure 4a).

A more complete theoretical work would involve, for example, performing molecular dynamics simulations of SERT complexes with chaetoglobosins and with SERT inhibitors, followed by calculations of the affinities of chaetoglobosins and inhibitors for SERTs using, for example, the molecular mechanics Poisson‐Boltzmann surface area (MMPBSA) method. However, the SERTs employed in this computational work are of human origin and exhibit approximately 53% identity with the nematode SERT. Therefore, to make the prediction based on computational calculations more robust, it would be necessary to model the nematode SERT three‐dimensionally, to redo the molecular docking and proceed to the molecular dynamics simulation and affinity calculations. However, the aim of this work was to employ simpler computational methods more accessible to the general public, to be carried out with simpler computers. Therefore, it was decided to continue the work by performing in vitro tests with SERT inhibitors and the nematode *M. incognita*.

### 2.5 Immobility and Mortality of *M. incognita* J2 after Exposure to SERT Inhibitors

**FIGURE 5:**
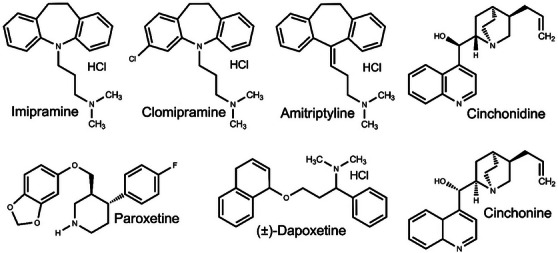
Chemical structures of sodium‐dependent serotonin transporter inhibitors that were tested against *Meloidogyne incognita* in the present work.

**FIGURE 6:**
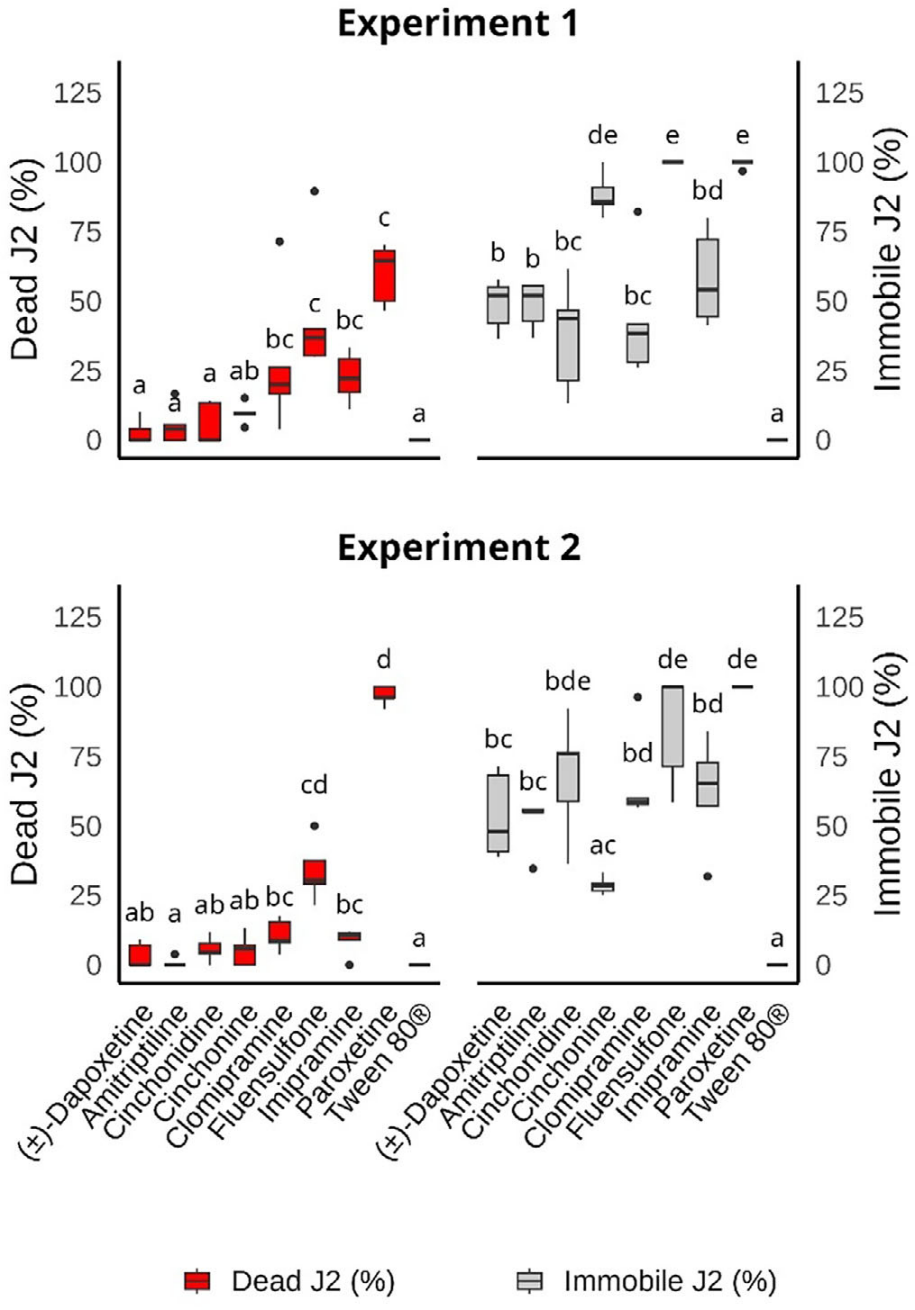
Boxplot of dead and immobile second‐stage juveniles (J2) of *Meloidogyne incognita* after 48 h exposure to different sodium‐dependent serotonin transporter inhibitors (amitriptyline, imipramine, (±)‐dapoxetine, clomipramine, paroxetine, cinchonine, and cichonidine). Fluensulfone and Tween 80 were used as positive and negative controls, respectively. Boxes of the same color, with at least one letter in common, in each graphic/experiment, are statistically equal to each other according to the Conover [28] test (*p* < 0.05; *n* = 45), with *p*‐value correction using the Bonferoni [29] method. Values are presented without any transformation.

All tested inhibitors (Figure 5) reduced the motility of *M. incognita*, with paroxetine causing the highest nematode mortality. Although imipramine, clomipramine, (±)‐dapoxetine, cinchonine, and cichonidine increased J2 mortality compared to the negative control, the percentages of dead J2 were no higher than 28% (Figure 6).

Paroxetine is a selective serotonin reuptake inhibitor (SSRI), used to treat depression, panic disorder, anxiety, post‐traumatic stress disorder, and social phobia among other conditions [30, 31]. However, no information is currently available regarding its nematicidal activity against *M. incognita*. The only existing data on nematocidal activity involve its in vitro effects on the free‐living nematode *C. elegans* [32].

When different concentrations of paroxetine were tested, its LC_50_ was determined to be 351.2 *µ*g/mL. Under the same conditions, fluensulfone had an LC_50_ of 39.31 *µ*g/mL, a value closely aligned with those reported in the literature for this commercial nematicide [33, 34]. Although the LC_50_ calculated for paroxetine is approximately 8.9 times higher than that of fluensulfone, it is important to note that fluensulfone is a commercial product that has undergone extensive optimization in order to maximize its efficacy against nematodes. In contrast, paroxetine has not yet been structurally optimized for nematode activity. Given this context, an LC_50_ only 8.9 times higher than that of a well‐established commercial nematicide is a highly promising result.

Serotonin is a neurotransmitter and neuromodulator involved in various functions of the free‐living nematode *C. elegans*, such as feeding, locomotion, egg laying, mating, and learning [35, 36, 37, 38, 39]. Exogenous serotonin has been demonstrated to stimulate stylet thrusting and reproductive behavior in several PPNs [15, 40, 41, 42, 43, 44, 45]. In the PPN *Heterodera glycines* Ichinohe, serotonin significantly inhibits body movement frequency and reduces the rate of egg hatching [45]. Moreover, inhibition of serotonin uptake into vesicles, has been shown to hinder serotonin‐induced stylet thrusting behavior in the PPN *Globodera pallida* Stone [15]. In the PPN *Pratylenchus penetrans* (Cobb) Filipjev & Schuurmans‐Stekhoven serotonin regulates the feeding and reproductive behaviors [42]. When RNA interference was employed to reduce serotonin synthesis in *Meloidogyne graminicola* Golden & Birchfield, nematode invasion, development, and reproduction were significantly reduced [46]. Therefore, it is expected that any compound that can inhibit SERT and thereby disrupt serotonin use can affect the nematode. This is the case, for example, with the SERT inhibitor paroxetine, which increased mortality in the free‐living nematode *C. elegans* [32]. Therefore, the increased mortality of *M. incongita* J2 in the present study, caused by paroxetine, seems entirely consistent with the knowledge described in the literature.

### 2.6 Effect of Paroxetine on the Pathogenicity of *M. incognita* J2 in Tomato Plants

**FIGURE 7:**
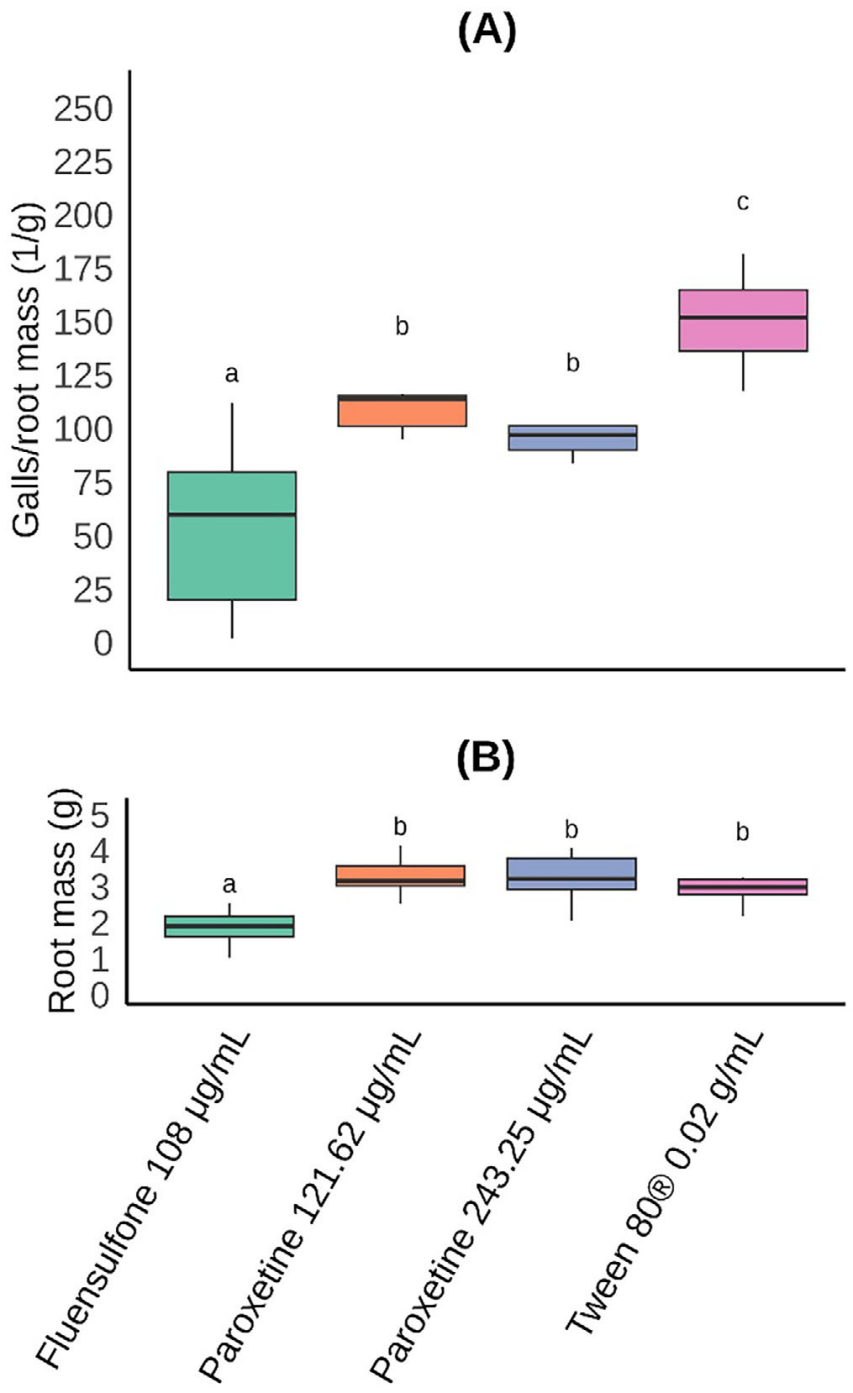
Boxplot of (a) galls per root mass (1/g) and (b) root mass (g) of tomato plants treated with paroxetine (two concentrations) and inoculated with *Meloidogyne incognita*. Fluensulfone (commercial nematicide) and Tween 80 were used as controls. Boxes with the same letter in each graphic are statistically equal to each other according to the Scott‐Knott [26] test (*p* < 0.05; *n* = 40), after ordered quantile transformation of data. Values are presented without any transformation.

At both tested concentrations, paroxetine treatment reduced the number of nematode galls (pathogenicity) per root mass compared to the negative control (Tween 80). Notably, both concentrations of paroxetine produced statistically equivalent results (Figure 7). Although the positive control (fluensulfone at 108 *µ*g/mL) was more effective in reducing nematode gall formation, it is important to consider that the root mass of plants treated with fluensulfone was statistically lower than that of the negative control. This significant root mass loss indicates the phytotoxic effects of this commercial nematicide on tomato plants. Since no phytotoxic effects were observed for paroxetine (Figure 7), this substance presents significant potential for structural optimization aimed at maximizing its nematicidal activity.

Although the commercial nematicide fluensulfone is more active against *M. incognita* than paroxetine, it is important to consider that this nematicide is approximately 50 times more expensive than paroxetine (https://www.molport.com). In addition, paroxetine has been used as a drug, which means that its toxicity to humans is much lower than that of fluensulfone. Furthermore, it should also be considered that fluensulfone is the result of extensive research aimed at maximizing nematicidal activity, while paroxetine can be the starting point for similar work, seeking to intensify nematicidal activity. Therefore, paroxetine presents great potential for use in the development of new commercial nematicides.

## 3 Conclusions

Computational calculations suggest that chaetoglobosins A and B act against *Meloidogyne* spp. by inhibiting the SERT produced by the nematodes. More advanced computational calculations and/or enzymatic tests should be performed in the future to corroborate this result. Paroxetine, which is a SERT inhibitor, reduced the mobility and increased the mortality of J2 of *M. incognita* in in vitro experiments. Additionally, it decreased the pathogenicity of the nematode in experiments in tomato seedlings trials. Therefore, studying SERT inhibitors – particularly compounds derived from paroxetine – holds significant potential for developing molecular structures aimed at controlling *M. incognita*.

## 4 Experimental Section

### 4.1 Preparation of Three‐Dimensional Structures of Chaetoglobosins

Using the Open3Dalign 2.3 computer program [47], the three‐dimensional structures of chaetoglobosins A and B (Figure 1) underwent conformational searches through molecular dynamics simulations, utilizing the Merck Molecular Force Field 94 (MMFF94). For each substance, 1000 molecular dynamics simulations were conducted, with 1000 steps of 1 fs per simulation. The most stable conformations, along with all those within 10 kcal/mol of the most stable, were then optimized using the Mopac 2016 program (http://openmopac.net/background.html). For this optimization, the PM7 Hamiltonian was applied, with the solvent (water) implicitly considered through the Conductor‐Like Screening Model (COSMO). Using the same program, the optimized structures underwent thermodynamic calculations to determine Gibbs free energy values. The Boltzmann distributions were then calculated for each chaetoglobosin conformation. Finally, using the computer program VMD 1.9.3 [48, 49], the root‐mean‐square deviation (RMSD) of atomic positions between each pair of conformations was calculated.

### 4.2 Virtual Screening

Only conformations representing 7% or more of the populations of chaetoglobosins A and B were used (Figure S1). The initial database utilized was Ligand Expo (http://ligand‐expo.rcsb.org/ld‐download.html) [50]. The search was conducted using the Lisica 1.0.1 computer program (http://insilab.org/lisica) [51]. Since all results had Tanimoto scores < 0.4, the search was expanded to the ChEMBL database (https://www.ebi.ac.uk) [52]. In this case, compounds with Tanimoto scores ≥ 0.4 were selected (Table S1).

### 4.3 Amino Acid Sequence Analysis

The amino acid sequences of the proteins inhibited by the compounds selected in Section above were used to conduct a search in the NCBI database (https://www.ncbi.nlm.nih.gov) [53] using the Blastp +2.13.0 computer program [54]. This search aimed to identify similar sequences within the genomes of *Meloidogyne* spp. (taxid:18290). Only proteins with a score above 200 were selected (Table S2).

### 4.4 Sodium‐Dependent Serotonin Transporter (SERT)

The 3D structures and amino acid sequences of the following SERTs (UniProtKB accession: P31645) were obtained from the RCSB Protein Data Bank (https://www.rcsb.org) [55]: 6VRH [17], 6VRK [17], 6VRL [17], 5I6X [23], 5I6Z [23], 5I66 [23], 5I73 [23], 5I74 [23], 5I75 [23]; 6AWN [24], 6AWP [24], 6AWQ [24], 6DZV [20], 6DZW [20], 6DZY [20], 6DZZ [20], 6W2C [17], 6W2B [17], 7LI7 [18], 7LI8 [18], 7LI9 [18], 7LIA [18], 7MGW [18], and 7LWD [19]. Using UGENE version 1.32.0 [56], with the Clustal Omega 1.2.1 algorithm [57], the amino acid sequences of all the SERTs were aligned in four iterations. Hamming dissimilarities (%) were calculated, accounting for all the gaps. Sequences with similarities (100 ‐ Hamming dissimilarity) of at least 86% compared to 6VRH [17] (Table S3) were selected. Antibodies not chemically bound to the SERTs were removed before performing the 3D alignment provided by Lovoalign 21.027 [21]. This program was also used to calculate the RMSD of atomic positions (Table S4) for each pair of 3D structures. Finally, mutant SERTs were discarded.

### 4.5 Molecular Docking

SDF files containing the three‐dimensional structures of SERT inhibitors were obtained from the RCSB Protein Data Bank (https://www.rcsb.org/) [55] (Table S5). Using the MarvinSketch 22.11 program (http://www.chemaxon.com), the protonation states of these inhibitors were calculated at pH 7. The structures of these inhibitors, the conformations of chaetoglobosins A and B (Figure S1), and the 3D structures of the biological units of the SERTs (Table S4), after alignment with the 6VRH [17] protein, were converted to PDBQT format using MGLTools Version 1.5.7rc1 [58]. The docking grid was centered at 133.85, 123.71, and 123.68 Å (x, y, and z), with dimensions of 24.31, 21.41, and 28.75 Å (x, y, and z) ensuring full coverage of the region where inhibitors were experimentally complexed to the SERTs. Using the QuickVina 2 program [25], the SERT inhibitors (Table S5) and the conformations of chaetoglobosins A and B (Figure S1) were docked to the SERTs (Table S6). Except for the exhaustiveness parameter, which was increased to 256, all other parameters remained at their default values. Overlap between the inhibitor 8PR (paroxetine) docked to the SERT 6VRH and the same inhibitor experimentally complexed to that SERT was analyzed using the UCSF Chimera 1.17.3 program (https://www.cgl.ucsf.edu/chimera/download.html) [22].

### 4.6 Obtaining Eggs and Second Stage Juveniles (J2) of *M. incognita*

Roots of tomato plants (*Solanum lycopersicum* L. ‘Santa Clara’) grown in a greenhouse and infested with *M. incognita* were thoroughly washed and cut into approximately 1 cm pieces. The roots were then ground in a blender for 30 s in a 0.5% (g/mL) sodium hypochlorite solution. The resulting suspension was filtered through two sieves with 200‐ and 500‐mesh pores. The eggs were washed thoroughly with water to remove any remaining sodium hypochlorite. Eggs retained on the 500‐mesh sieve were collected and placed in a hatching chamber at a fixed temperature of 28°C to obtain second‐stage juveniles (J2). Any J2 hatched within the first 24 h were discarded. Only those hatched within a maximum of 48 h before the experiment setup were used in the tests.

### 4.7 *M. incognita* J2 Mobility and Mortality Test

In 96‐well microplates, 20 *µ*L of an aqueous suspension containing approximately 20 J2 of *M. incognita* and 100 *µ*L of each SERT inhibitor solution (Figure 5) were added. These solutions were prepared by dissolving the inhibitors in an aqueous Tween 80 (Sigma‐Aldrich, MO, USA) solution at 0.02 g/mL, up to a concentration of 600 *µ*g/mL. The inhibitor suspensions were stirred using a magnetic stirrer and subjected to ultrasound until they became clear and homogeneous. The microplates were kept in a BOD at 28°C. The experiment was conducted in five replicates, using fluensulfone (Nimitz, ADAMA) at 34 *µ*g/mL (final concentration after adding the J2 suspension) as a positive control. A Tween 80 solution at 0.02 g/mL served as the negative control. After 48 h, mobile and immobile J2 were counted under a microscope. A freshly prepared 1.0 mol/L NaOH solution (5 *µ*L) was then added to each cavity, and J2 were counted again for up to 1 min after NaOH addition. J2 that did not respond to the NaOH solution and remained straight and immobile were classified as dead, while those that exhibited movement and twisting were considered alive, following the method described by Chen and Dickson [59], as adapted by Amaral et al. [60]. This experiment was performed twice.

### 4.8 Lethal Concentrations for 50% (LC_50_) of *M. incognita* J2

The procedure used was the same as described in Section above to determine J2 mortality. The final concentrations in the wells, after mixing with the J2 suspension, were 200, 250, 300, 350, and 400 *µ*g/mL for paroxetine (Figure 5, purity: ≥ 98%; Origin: Cayman Chemical) and 25, 30, 35, 40, and 45 *µ*g/mL for fluensulfone (Nimitz, ADAMA, USA). This experiment was conducted three times.

### 4.9 Effect of Paroxetine on the Pathogenicity of *M. incognita* in Tomato Plants

Based on the literature [61], tomato seeds (*Solanum lycopersicum* L. ‘Santa Clara’) susceptible to *M. incognita* were sown in the commercial substrate Tropstrato (Vida Verde Indústria e Comércio de Insumos Orgânicos Ltda., Mogi Mirim, São Paulo, Brazil) contained in Styrofoam seed cells, with 72 cells of 121 mL each. Paroxetine (Purity: ≥ 98%; Cayman Chemical) was dissolved in an aqueous Tween 80 solution (0.02 g/mL) to concentrations of 243.25 and 121.62 *µ*g/mL. An aqueous suspension (5.6 mL) containing approximately 500 J2 of *M. incognita* was applied to four holes (0.4 cm wide and 1.5 deep) around the stem of each 25‐day‐old tomato plant (1.4 mL per hole). Immediately afterwards, 2 mL of one of the solutions of paroxetine were applied separately to the same holes (0.5 mL per hole). Tween 80 at 0.02 g/mL and fluensulfone (Nimitz, ADAMA, USA) at 108.89 *µ*g/mL, dissolved in water, were used as negative and positive controls, respectively. The seedlings were kept in a shaded room at approximately 28°C for 48 h before being transferred to a greenhouse, where they remained for approximately 45 days. After this period, the roots were removed, washed with water, dried with paper towels, and subjected to the nematode galls count. The experiment was conducted twice.

### 4.10 Statistical Analysis

The affinity values obtained from the docking procedure were subjected to the Shapiro‐Wilk [62] normality test and Barlett's [63] homoscedasticity test. Since both tests were satisfied (*p* > 0.5; *n* = 56), analysis of variance (ANOVA) was carried out (*p* < 0.05; *n* = 56), and the means were compared using the Scott‐Knott [26] grouping test at 5% significance (*n* = 56). The data from the *M. incognita* J2 mobility and mortality test were separately (data from each experiment and parameter underwent separate calculations) converted into percentages and subjected to the Shapiro‐Wilk [62] normality test and Barlett's [63] homoscedasticity test. Despite multiple transformations, normalization and homoscedasticity were not achieved (*p* < 0.05; n = 45). Consequently, a non‐parametric Kruskal‐Wallis [64] test was performed, revealing that at least one treatment differed significantly from the others (*p* < 0.01; *n* = 45). The Conover [28] test was then applied at a 5% significance level (*n* = 45), using the Bonferoni [29] method to adjust *p*‐values. The LC_50_ values were calculated through logit analyses [65], performed with the drc package [66]. To verify whether the obtained curves satisfactorily fit the experimental data, the lack‐of‐fit test was employed [67] (*p* > 0.07; *n* = 30). When combined, data from both pathogenicity of *M. incognita* in tomato plants experiments did not pass the Shapiro‐Wilk [62] normality test or Barlett's [63] homoscedasticity test (*p* < 0.05; *n* = 40). Consequently, they were transformed using the Ordered Quantile [68] method, which enabled normalization and homoscedasticity (*p* > 0.2; n = 40). Two‐way ANOVA revealed no interaction between the independent variables, experiment repetition, or additive effects (*p* > 0.1; *n* = 40). The Scott‐Knott [26] test was then performed at a 5% significance level (*n* = 40). All analyses were conducted using the R program, version 4.4.0 [69, 70].

## Author Contribution

**Denilson F. de Oliveira**: Writing – original draft, methodology, funding acquisition, formal analysis, data curation, conceptualization. **Geraldo J. Hodela**: Writing – original draft, methodology, formal analysis, conceptualization. **Vitor P. de Sousa**: Writing – original draft, methodology. **Mariana C. de Melo**: Writing – original draft, methodology. **Rodrigo M. Fráguas**: Writing – original draft, methodology. **Willian C. Terra**: Writing – original draft, methodology.

## Conflicts of Interest

The authors declare no conflicts of interest.

## Supporting information




**Supplementary File 1**: cbdv71122‐sup‐0001‐SuppMat.docx

## Data Availability

The data that support the findings of this study are available in the supplementary material of this article.;
