# Supplementary material for: Inhibitors of Sodium‐Dependent Serotonin Transporter Protein (SERT) as Potential New Nematicides
Source: Chem Biodivers. 2026 Mar 26;23(3):e03443. doi: 10.1002/cbdv.202503443 (PMC13022468; doi:10.1002/cbdv.202503443)
Supplement: Supplementary file 1 — cbdv71122‐sup‐0001‐SuppMat.docx [file CBDV-23-e03443-s001.docx]

**Supporting Information**

Inhibitors of Sodium-Dependent Serotonin Transporter Protein (SERT) as Potential New Nematicides

Geraldo Jamisse Hodela,^a^ Vitor Pereira de Sousa, ^b^  Mariana Castro de Melo, ^a^ Rodrigo Martins Fráguas, ^a^ Willian César Terra ^b^ and Denilson Ferreira de Oliveira*,^a^

^a^ Department of Chemistry, Federal University of Lavras, P.O. BOX 3037, Lavras-37203-202, MG-Brazil

^b^ Department of Phytopathology, Federal University of Lavras, P.O. Box 3037, Lavras-37.203-202, MG-Brazil

*Corresponding author :Denilson F. Oliveira **(**[denilson@ufla.br](mailto:denilson@ufla.br))


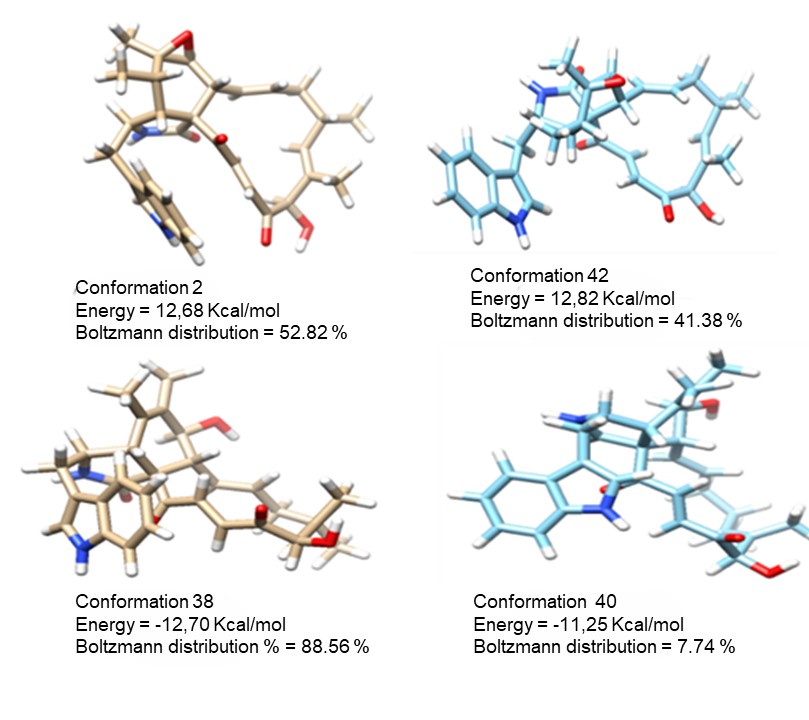


Figure S1 | Most stable conformations for chaetoglobosins A (conformation 2 and 42) and B (conformation 38 and 40).

Table S1 | Codes of substances selected in the three-dimensional search in the ChEMBL[1] database for chemical structures similar to chaetoglobosins A and B.

| **Substance code** | **Tanimoto score** | **Protein on which the substance acts** | **Protein producing organism** | **Reference** |
| --- | --- | --- | --- | --- |
| CHEMBL606244 | 0.40 | Sodium-dependent serotonin transporter (SERT) | *Homo sapiens* | [2] |
| CHEMBL2138465 | 0.40 | Glutaminase | *Homo sapiens* | [3] |
| CHEMBL2138465 | 0.40 | *Ampc-β-*lactamase | *Escherichia coli* | [4] |

Table S2 | Substance and protein code according to the search carried out in the ChEMBL database. Protein name of the amino acid sequence detected in the genome of *Meloidogyne graminicola*, followed by score, coverage and identity with respect to the amino acid sequence of the protein code.

| **Substance code** | **Protein code** | **Protein name** | **Score** | **Coverage** | **Identity** |
| --- | --- | --- | --- | --- | --- |
| CHEMBL606244 | P31645 | Sodium-dependent serotonin transporter (SERT) | 610 | 81% | 53.28% |

Table S3 | Amino acid sequence similarity (%) of sodium-dependent serotonin transporters (SERTs), calculated by Ugene 1.32.0[5], after alignment with Clustal Omega 1.2.4 through four iterations[6] .

| **Protein codes** | **Producing organism** | **Amino acid residues** | **P31645^a^ (*Homo sapiens*)** | **6VRH^a^ (*Homo sapiens*)** | **6VRK^a^ (*Homo sapiens*)** | **6VRL^a^ (*Homo sapiens*)** |
| --- | --- | --- | --- | --- | --- | --- |
| P31645^a^ | *Homo sapiens* | 630 | 100 | 100 | 100 | 100 |
| 5I6X^a^ | *Homo sapiens* | 549 | 87 | 87 | 87 | 87 |
| 5I6Z^a^ | *Homo sapiens* | 549 | 87 | 87 | 87 | 87 |
| 5I71^a^ | *Homo sapiens* | 549 | 87 | 87 | 87 | 87 |
| 5I73^a^ | *Homo sapiens* | 549 | 87 | 87 | 87 | 87 |
| 5I74^a^ | *Homo sapiens* | 549 | 87 | 87 | 87 | 87 |
| 5I75^a^ | *Homo sapiens* | 549 | 87 | 87 | 87 | 87 |
| 6AWN^a^ | *Homo sapiens* | 549 | 87 | 87 | 87 | 87 |
| 6AWO^a^ | *Homo sapiens* | 549 | 87 | 87 | 87 | 87 |
| 6AWP^a^ | *Homo sapiens* | 549 | 87 | 87 | 87 | 87 |
| 6AWQ^a^ | *Homo sapiens* | 549 | 87 | 87 | 87 | 87 |
| 6DZV^a^ | *Homo sapiens* | 537 | 86 | 86 | 86 | 86 |
| 6DZW^a^ | *Homo sapiens* | 537 | 86 | 86 | 86 | 86 |
| 6DZY^a^ | *Homo sapiens* | 537 | 86 | 86 | 86 | 86 |
| 6DZZ^a^ | *Homo sapiens* | 540 | 87 | 87 | 87 | 87 |
| 6VRH^a^ | *Homo sapiens* | 630 | 100 | 100 | 100 | 100 |
| 6VRK^a^ | *Homo sapiens* | 630 | 100 | 100 | 100 | 100 |
| 6VRL^a^ | *Homo sapiens* | 630 | 100 | 100 | 100 | 100 |
| 6W2B^a^ | *Homo sapiens* | 549 | 87 | 87 | 87 | 87 |
| 6W2C^a^ | *Homo sapiens* | 549 | 87 | 87 | 87 | 87 |
| 7LI6^a^ | *Homo sapiens* | 539 | 87 | 87 | 87 | 87 |
| 7LI7^a^ | *Homo sapiens* | 537 | 86 | 86 | 86 | 86 |
| 7LI8^a^ | *Homo sapiens* | 539 | 87 | 87 | 87 | 87 |
| 7LI9^a^ | *Homo sapiens* | 539 | 87 | 87 | 87 | 87 |
| 7LIA^a^ | *Homo sapiens* | 539 | 87 | 87 | 87 | 87 |
| 7LWD^a^ | *Homo sapiens* | 541 | 87 | 87 | 87 | 87 |
| 7MGW^a^ | *Homo sapiens* | 537 | 86 | 86 | 86 | 86 |

____________________________

^a^ Sodium- dependent serotonin transporters (SERT).

Table S4 | Codes for the three-dimensional structures of the biological units of SERTs, after alignment with the protein 6VRH[7], using the Lovoalign computer programme version 21.027[8], which was also used to calculate root mean square deviation of atomic positions (RMSD) in relation to 6VRH.

| Protein codes | Amino acid residues | RMSD (Å) |
| --- | --- | --- |
| 5I6X | 549 | 1.096 |
| 5I6Z | 549 | 1.089 |
| 5I71 | 549 | 1.145 |
| 5I73 | 549 | 1.156 |
| 5I74 | 549 | 1.145 |
| 5I75 | 549 | 1.164 |
| 6AWN | 549 | 1.113 |
| 6AWO | 549 | 1.111 |
| 6AWP | 549 | 1.116 |
| 6AWQ | 549 | 1.111 |
| 6DZV | 537 | 1.801 |
| 6DZW | 537 | 1.275 |
| 6DZY | 537 | 1.202 |
| 6DZZ | 540 | 4.065 |
| 6VRH | 630 | 0.000 |
| 6VRK | 630 | 0.626 |
| 6VRL | 630 | 0.589 |
| 6W2B | 549 | 1.114 |
| 6W2C | 549 | 1.114 |
| 7LI6 | 539 | 4.679 |
| 7LI7 | 537 | 1.740 |
| 7LI8 | 539 | 4.894 |
| 7LI9 | 539 | 4.160 |
| 7LIA | 539 | 0.990 |
| 7LWD | 541 | 1.028 |
| 7MGW | 537 | 1.822 |

**Table S5** **|** Codes and names of sodium-dependent serotonin transporter (SERT) inhibitors.

| **Ligand codes** | **Ligand names** | **References** |
| --- | --- | --- |
| 8PR | (3*S*,4*R*)-3-(1,3-benzodioxol-5-yloxymethyl)-4-(4-fluorophenyl)piperidine | [9] |
| 68P | **(1*S*)-1-[3-(dimethylamino)propyl]-1-(4-fluorophenyl)-1,3-dihydro-2-benzofuran-5-carbonitrile** | [9] |
| 69D | **(1*S*)-1-(4-bromophenyl)-1-[3-(dimethylamino)propyl]-1,3-dihydro-2-benzofuran-5-carbonitrile** | [9] |
| 8PR | (3*S*,4*R*)-3-[(1,3-benzodioxol-5-yloxy) methyl]-4-(4-fluorophenyl) piperidine | [10] |
| FVX | 2-[(*E*)-[5-methoxy-1-[4 (triflouromethyl)phenyl]pentylidene]amino]oxyethanamine | [10] |
| SRE | **(1*S*,4*S*)-4-(3,4-dichlorophenyl)-*N*-methyl-1,2,3,4-tetrahydronaphthalen-1-amine** | [10] |

Table S6 | Codes for the three-dimensional structures of sodium-dependent serotonin transporters (SERTs) that were selected for the molecular docking.

| **Protein codes** | **Producing species** | **Number of amino acid residues** | **References** |
| --- | --- | --- | --- |
| 7MGW | [*Homo sapiens*](https://www.rcsb.org/search?q=rcsb_entity_source_organism.taxonomy_lineage.name:Homo%20sapiens) | 537 | [11] |
| 7LI7 | [*Homo sapiens*](https://www.rcsb.org/search?q=rcsb_entity_source_organism.taxonomy_lineage.name:Homo%20sapiens) | 537 | [11] |
| 7LWD | [*Homo sapiens*](https://www.rcsb.org/search?q=rcsb_entity_source_organism.taxonomy_lineage.name:Homo%20sapiens) | 541 | [12] |
| 7LIA | [*Homo sapiens*](https://www.rcsb.org/search?q=rcsb_entity_source_organism.taxonomy_lineage.name:Homo%20sapiens) | 539 | [11] |
| 7LI9 | [*Homo sapiens*](https://www.rcsb.org/search?q=rcsb_entity_source_organism.taxonomy_lineage.name:Homo%20sapiens) | 539 | [11] |
| 7LI8 | [*Homo sapiens*](https://www.rcsb.org/search?q=rcsb_entity_source_organism.taxonomy_lineage.name:Homo%20sapiens) | 539 | [11] |
| 6DZZ | [*Homo sapiens*](https://www.rcsb.org/search?q=rcsb_entity_source_organism.taxonomy_lineage.name:Homo%20sapiens) | 540 | [13] |
| 7LI6 | [*Homo sapiens*](https://www.rcsb.org/search?q=rcsb_entity_source_organism.taxonomy_lineage.name:Homo%20sapiens) | 539 | [13] |
| 6VRL | [*Homo sapiens*](https://www.rcsb.org/search?q=rcsb_entity_source_organism.taxonomy_lineage.name:Homo%20sapiens) | 630 | [13] |
| 6VRK | [*Homo sapiens*](https://www.rcsb.org/search?q=rcsb_entity_source_organism.taxonomy_lineage.name:Homo%20sapiens) | 630 | [13] |
| 6VRH | [*Homo sapiens*](https://www.rcsb.org/search?q=rcsb_entity_source_organism.taxonomy_lineage.name:Homo%20sapiens) | 630 | [13] |
| 6DZV | [*Homo sapiens*](https://www.rcsb.org/search?q=rcsb_entity_source_organism.taxonomy_lineage.name:Homo%20sapiens) | 537 | [13] |

**References**

[1] D. Mendez, A. Gaulton, A. P. Bento, J. Chambers, M. De Veij, E. Félix, M. P. Magariños, J. F. Mosquera, P. Mutowo, M. Nowotka, ^ʺ^ChEMBL: towards direct deposition of bioassay dataʺ, Nucleic Acids Research 47 (2019): D930–D940, [*https://doi.org/10.1093/nar/gky1075*](https://doi.org/10.1093/nar/gky1075)*.*

[2] L. R. Marcin, R. J. Mattson, Q. Gao, D. Wu, T. F. Molski, G. K. Mattson, N. J. Lodge, ^ʺ^Synthesis and hSERT activity of homotryptamine analogs. Part 6:[3+ 2] dipolar cycloaddition of 3-vinylindolesʺ, *Bioorganic & Medicinal Chemistry Letters* 20 (2010):1027–1030, <https://doi.org/10.1016/j.bmcl.2009.12.043>.

[3] S. C. Zimmermann, E. F. Wolf, A. Luu, A. G. Thomas, M. Stathis, B. Poore, C. Nguyen, A. Le, C. Rojas, B. S. Slusher, ʺAllosteric glutaminase inhibitors based on a 1, 4-di (5-amino-1, 3, 4-thiadiazol-2-yl) butane scaffoldʺ, *ACS medicinal chemistry letters* 7 (2016): 520–524, <https://doi.org/10.1021/acsmedchemlett.6b00060>.

[4] V. Buzzoni, J. Blazquez, S. Ferrari, S. Calo, A. Venturelli, M. P. Costi, ʺAza-boronic acids as non-β-lactam inhibitors of AmpC-β-lactamaseʺ, *Bioorganic & medicinal chemistry letters* 14 (2004): 3979–3983, <https://doi.org/10.1016/j.bmcl.2004.05.054>.

[5] K. Okonechnikov, O. Golosova, M. Fursov, A. Varlamov, Y. Vaskin, I. Efremov, O. G. German Grehov, D. Kandrov, K. Rasputin, M. Syabro, T. Tleukenov. ʺUnipro UGENE: a unified bioinformatics toolkitʺ, *Bioinformatics* 28 (2012) 1166–1167, <https://doi.org/10.1093/bioinformatics/bts091>.

[6] F. Sievers, D. G. Higgins, ^ʺ^Clustal Omega for making accurate alignments of many protein sequences^ʺ^, *Protein Science* 27 (2018): 35–145, <https://doi.org/10.1002/pro.3290>.

[7] J. A. Coleman, V. Navratna, D. Antermite, D. Yang, J. A. Bull, E. Gouaux, ^ʺ^Chemical and structural investigation of the paroxetine-human serotonin transporter complexʺ, *Elife* 9 (2020): e56427, <https://doi.org/10.7554/elife.56427>.

[8] L. Martínez, R. Andreani, J. M. Martínez, ʺConvergent algorithms for protein structural alignmentʺ, *BMC Bioinformatics* 8 (2007), <https://doi.org/10.1186/1471-2105-8-306>.

[9] J. A. Coleman, E. M. Green, E. Gouaux, ʺX-ray structures and mechanism of the human serotonin transporterʺ, *Nature* 532 (2016):334–339, <https://doi.org/10.1038/nature17629>.

[10] J. A. Coleman, E. Gouaux, ʺStructural basis for recognition of diverse antidepressants by the human serotonin transporterʺ *Nature Structural & Molecular Biology* 25 (2018) 170–175, <https://doi.org/10.1038/s41594-018-0026-8>.

[11] D. Yang, E. Gouaux, ^ʺ^Illumination of serotonin transporter mechanism and role of the allosteric siteʺ, Science Advances 7 (2021): eabl3857. <https://doi.org/10.1126/sciadv.abl3857>.

[12] P. Plenge, D. Yang, K. Salomon, L. Laursen, I. E. Kalenderoglou, A. H. Newman, E. Gouaux, J. A. Coleman, C. J. Loland, ʺThe antidepressant drug vilazodone is an allosteric inhibitor of the serotonin transporterʺ, *Nature Communications* 12 (2021): 5063, <https://doi.org/10.1038/s41467-021-25363-3>.

[13] J. A. Coleman, D. Yang, Z. Zhao, P.-C. Wen, C. Yoshioka, E. Tajkhorshid, E. Gouaux, ʺSerotonin transporter–ibogaine complexes illuminate mechanisms of inhibition and transport ʺ, *Nature* 569 (2019): 141–145, <https://doi.org/10.1038/s41586-019-1135-1>.
